# Parp1 protects against Aag-dependent alkylation-induced nephrotoxicity in a sex-dependent manner

**DOI:** 10.18632/oncotarget.10440

**Published:** 2016-07-06

**Authors:** Jennifer A. Calvo, Mariacarmela Allocca, Kimberly R. Fake, Sureshkumar Muthupalani, Joshua J. Corrigan, Roderick T. Bronson, Leona D. Samson

**Affiliations:** ^1^ Department of Biological Engineering, Massachusetts Institute of Technology, Cambridge, MA, USA; ^2^ Center for Environmental Health Sciences, Massachusetts Institute of Technology, Cambridge, MA, USA; ^3^ Division of Comparative Medicine, Massachusetts Institute of Technology, Cambridge, MA, USA; ^4^ Rodent Histopathology Core, Harvard Medical School, Boston, MA, USA; ^5^ The David H. Koch Institute for Integrative Cancer Research Massachusetts Institute of Technology, Cambridge, MA, USA

**Keywords:** alkylating agents, MMS, Parp1, Aag, nephrotoxicity, Pathology Section

## Abstract

Nephrotoxicity is a common toxic side-effect of chemotherapeutic alkylating agents. Although the base excision repair (BER) pathway is essential in repairing DNA alkylation damage, under certain conditions the initiation of BER produces toxic repair intermediates that damage healthy tissues. We have shown that the alkyladenine DNA glycosylase, Aag (a.k.a. Mpg), an enzyme that initiates BER, mediates alkylation-induced whole-animal lethality and cytotoxicity in the pancreas, spleen, retina, and cerebellum, but not in the kidney. Cytotoxicity in both wild-type and *Aag*-transgenic mice (*AagTg*) was abrogated in the absence of Poly(ADP-ribose) polymerase-1 (*Parp1*). Here we report that Parp1-deficient mice expressing increased Aag (*AagTg/Parp1^−/−^)* develop sex-dependent kidney failure upon exposure to the alkylating agent, methyl methanesulfonate (MMS), and suffer increased whole-animal lethality compared to *AagTg* and wild-type mice. Macroscopic, histological, electron microscopic and immunohistochemical analyses revealed morphological kidney damage including dilated tubules, proteinaceous casts, vacuolation, collapse of the glomerular tuft, and deterioration of podocyte structure. Moreover, mice exhibited clinical signs of kidney disease indicating functional damage, including elevated blood nitrogen urea and creatinine, hypoproteinemia and proteinuria. Pharmacological Parp inhibition in *AagTg* mice also resulted in sensitivity to MMS-induced nephrotoxicity. These findings provide *in vivo* evidence that Parp1 modulates Aag-dependent MMS-induced nephrotoxicity in a sex-dependent manner and highlight the critical roles that Aag-initiated BER and Parp1 may play in determining the side-effects of chemotherapeutic alkylating agents.

## INTRODUCTION

The kidney is essential for excretion of waste and maintenance of proper osmotic and oncotic pressure; these functions require a properly functioning filtration unit comprised of the glomerulus and surrounding Bowman's capsule. Blood is filtered within the filtration unit; protein, cells, and specific concentrations of chemicals are retained, whereas waste and extra fluid is passed to the renal tubules to become urine. One common feature of glomerular dysfunction is loss of protein from the blood (hypoproteinemia) accompanied by increased urinary protein (proteinuria). Glomerular dysfunction can cause chronic kidney disease and eventually end-stage renal disease (ESRD), requiring dialysis or kidney transplantation. Various causes include genetic mutations, autoimmunity, infections, environmental exposures or any combination thereof (reviewed in [1–4]). Pathological changes damaging the glomerular filtration apparatus are responsible for 90% of ESRD, resulting in ~$20 billion in yearly health costs in the USA [5].

The glomerular filtration unit has three layers: the visceral epithelial cells (podocytes), the glomerular basement membrane, and parietal endothelial cells; proper communication and function of all three components is required for glomerular function. Podocytes, the most essential cell-type for glomerular filtration, possess interdigitating foot processes that form filtration slits called slit diaphragms (reviewed in [6]). Loss of the slit diaphragm through effacement of the podocyte foot processes is sufficient to cause proteinuria and kidney damage, emphasizing the importance of podocytes in proper kidney function [7]. Various podocytopathies play a dominant role in glomerular diseases [2].

Some cancer chemotherapeutic agents, including alkylating agents, induce nephrotoxicity. For example, oxazaphosphorines, used in the treatment of certain leukemias, lymphomas and solid tumors, result in significant nephrotoxicity [8–10]. Such off-target toxicity is dose-limiting, thus reducing efficacy.

Alkylating agents represent one class of commonly-utilized chemotherapeutic agents that generate numerous types of alkylated DNA base lesions, including *O*^6^-alkylguanine (*O*^6^alkG), 7-alkylguanine (7alkG) and 3alkyladenine (3alkA). The induction of cell death in rapidly-dividing cells by these toxic DNA lesions underlies the effectiveness of alkylators as cancer chemotherapeutic agents. The base excision repair (BER) pathway repairs many alkylated DNA base lesions [11]. BER is initiated by the recognition and excision of alkylated DNA base lesions by the alkyladenine DNA glycosylase, Aag (a.k.a. Mpg). After base excision, an AP endonuclease (Ape1) hydrolyzes the phosphodiester backbone at the abasic site, generating a single-stranded DNA break (SSB) with 3′OH and 5′deoxyribose-5-phosphate (5′dRP) termini. Poly(ADP-ribose) polymerase-1 (Parp1) acts as a SSB sensor, and, upon binding a SSB, is activated to catalyze the addition of long polymers of ADP-ribose to itself and several other proteins. The poly(ADP-ribose) chains are thought to recruit downstream BER enzymes: DNA polymerase β (Pol β) that removes the 5′dRP terminus and extends the missing nucleotide from the 3′OH, and either DNA Ligase I or the Xrcc1/LigaseIIIα complex that seal the remaining nicked DNA to complete BER.

Although BER can repair DNA alkylation damage, under certain conditions the initiation of BER can generate toxic repair intermediates that cause damage to healthy tissues. Recently, using mouse genetic models, we demonstrated the importance of both Aag and Parp1 in modulating *in vivo* alkylation sensitivity. Modest increases in Aag activity in a transgenic mouse model (*AagTg* mice) increased susceptibility to the alkylating agent, methyl methanesulfonate (MMS), for both whole-animal survival and tissue damage in a specific subset of tissues [12, 13]. The Aag-mediated alkylation sensitivity of these tissues, for both wild-type (WT) and *AagTg* mice, is entirely Parp1-dependent, being wholly prevented by Parp1 deficiency. Here, we show that although Parp1 deficiency protects against Aag-dependent, MMS-mediated tissue degeneration in the pancreas, spleen, retina, and cerebellum [12], Parp1 deficiency is surprisingly unable to protect against MMS-mediated whole-animal lethality. In fact, *AagTg/Parp1^−/−^* mice exhibit greater morbidity and lethality following MMS treatment as compared to *AagTg* mice. Strikingly, following MMS treatment, *AagTg/Parp1^−/−^* mice exhibit severe glomerular damage, suggesting a novel role for Aag and Parp1 in modulating the renal toxicity of alkylating agents.

Numerous animal models of acquired podocyte diseases have been described (reviewed in [14, 15]). MMS-treated *AagTg*/*Parp1^−/−^* mice represent a new model of glomerular disease and may provide insight into many types of human glomerular diseases. Further, the induction of nephrotoxicity by MMS is novel and the importance of Parp1 and Aag in modulating responses is especially relevant, given the recent surge in the use of PARP inhibitors for multiple clinical indications and the wide range of AAG activity in the human population [12, 16, 17].

## RESULTS

### *AagTg* and *Parp1^−/−^* alleles display synthetic lethality for alkylation-induced animal toxicity

*AagTg* mice, expressing higher than WT levels of Aag, display increased sensitivity to MMS toxicity compared to WT mice, at the whole-animal level and in numerous tissues including the cerebellum, retina, and pancreas, but not kidney [12, 13]. However, Parp1 deficiency completely suppresses MMS-mediated damage to these tissues in both WT and *AagTg* mice [12]. To determine whether Parp1 deficiency also protects against whole-body alkylation sensitivity in *AagTg* mice, we determined the LD_50_ of the alkylating agent MMS in WT, *Parp1^−/−^*, *AagTg*, and *AagTg*/*Parp1^−/−^* mice. Surprisingly, although alkylation-induced damage in numerous tissues is suppressed in *AagTg*/*Parp1^−/−^* mice [12], whole-animal toxicity is not suppressed; rather, the animals are more susceptible to MMS-induced whole-animal lethality. The MMS LD_50_ for *AagTg* mice is 80 mg/kg as we previously reported [12], and 66 mg/kg for *AagTg*/*Parp1^−/−^* mice (Table 1). *Parp1^−/−^* mice exhibit the same whole-body sensitivity to MMS as WT (LD_50_ 200 mg/kg). Remarkably, during a 30-day (d) survival experiment post-MMS treatment (75 mg/kg, a dose slightly below the LD_50_ of *AagTg* mice), we observed that *AagTg/Parp1^−/−^* mice began to lose significant body weight (Figure 1A) and exhibited signs of severe disease, including lethargy, a hunched posture, and cachexia (losing ~20% of initial BW) by 15d post-MMS treatment*;* all *AagTg/Parp1^−/−^* mice succumb within 30d (Figure 1B-1C).

**Table 1:** Approximate LD_50_ of MMS in mutant mice

	Approximate LD50^#^
Mouse Strain	MMS
WT	200 mg/kg
*Parp1^−/−^*	200 mg/kg
*AagTg*	80 mg/kg
*AagTg/Parp1^−/−^*	66 mg/kg

# as determined by Deichmann and LeBlanc method (1943). MMS, methyl methanesulfonate

**Figure 1:**
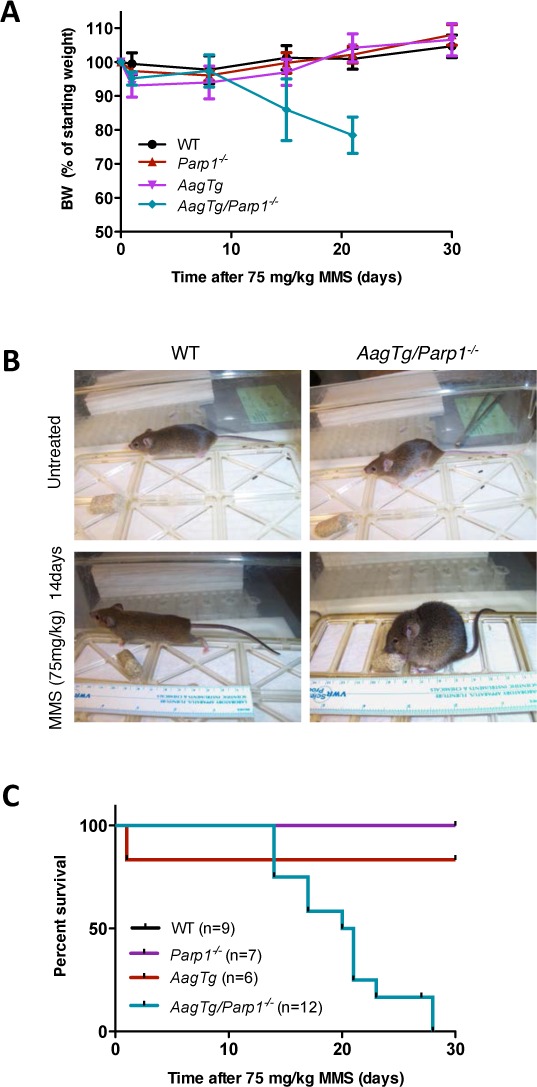
Parp1 deficiency results in increased overall whole-body sensitivity to MMS toxicity **A.** Body weight, as percentage of initial body weight following MMS treatment (75 mg/kg), is illustrated for WT (*n* = 17), *Parp1^−/−^* (*n* = 9), *AagTg* (*n* = 19), and *AagTg/Parp1^−/−^* (*n* = 22) mice at different time points. Representative data from two experiments are shown. **B.** Representative pictures of mouse body condition 14 days post-MMS treatment (75 mg/kg). **C.** Kaplan Meier survival curves are shown for WT (*n* = 9), *Parp1^−/−^* (*n* = 7), *AagTg* (*n* = 6), and *AagTg/Parp1^−/−^* (*n* = 12) mice following treatment with MMS (75 mg/kg).

### *AagTg* mice combined with either the *Parp1^−/−^* allele or Parp inhibition are susceptible to MMS-mediated renal toxicity

**Figure 2:**
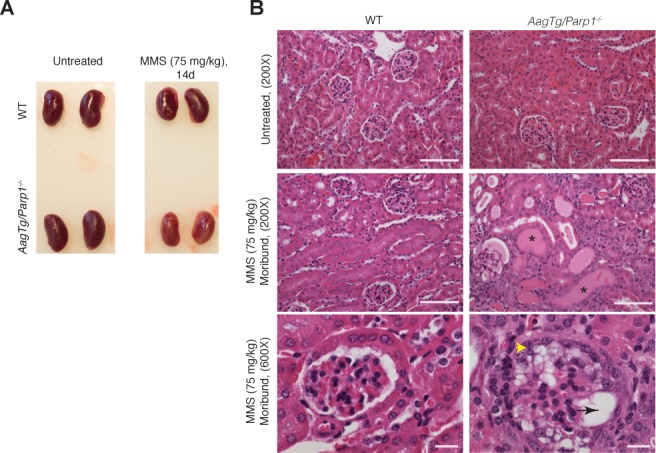
*AagTg/Parp1^−/−^* mice exhibit MMS-mediated kidney damage **A.** Representative images of kidneys following MMS treatment. Kidneys from a moribund *AagTg/Parp1^−/−^* mouse and a WT mouse 14 days post-MMS treatment (75 mg/kg) are shown. **B.** H&E analysis of kidneys from WT, *AagTg*, *Parp1^−/−^*, and *AagTg*/*Parp1^−/−^* mice 14 days following MMS treatment (75 mg/kg). Magnification is 200X (scale bar 50 μm) and 600x (scale bar 10 μm); asterisks indicate proteinaceous casts, the black arrow indicates vacuolation, and yellow arrowhead indicates parietal and visceral epithelial hypertrophy/hypercellularity with vacuolation, tuft adhesions, capillary collapse and crescent formation.

To investigate *causa mortis* in *AagTg/Parp1^−/−^* mice, we examined a panel of tissues at gross and histological levels 14d post-MMS. Remarkably, only the kidneys displayed alkylation-induced pathology. MMS-treated *AagTg/Parp1^−/−^* kidneys were smaller and paler compared to untreated and MMS-treated WT mice (Figure 2A). Accordingly, severe histological changes were observed. Under low magnification, we observed large, pink, proteinaceous casts that resulted in dilation of the kidney tubules (Figure 2B). High magnification revealed severe glomerular abnormalities, including vacuolation and loss of the Bowman's space (Figure 2B). Periodic acid-Schiff (PAS) staining shows a glomerular structure severely disturbed in *AagTg/Parp1^−/−^* mice post-MMS, as evident by vacuolation and glomerular tuft collapse (Supplemental Figure 1). No evidence of kidney disease was seen in MMS-treated WT, *AagTg* (Figure 2B and Supplemental Figure 1) or *Parp1^−/−^* (not shown) mice. Moreover, no other tissues from MMS-treated *AagTg/Parp1^−/−^* mice revealed histological abnormalities (not shown) at 14d. *AagTg/Parp1^−/−^* kidneys stained with PAS, toludine blue and phosphotungstic acid-hematoxilin (PTAH) 14d post-MMS showed no evidence of abnormal glomerular cellular accumulations, intravascular fibrin thrombi, matrix deposits, or significant fibrosis (Supplemental Figure 2).

**Figure 3:**
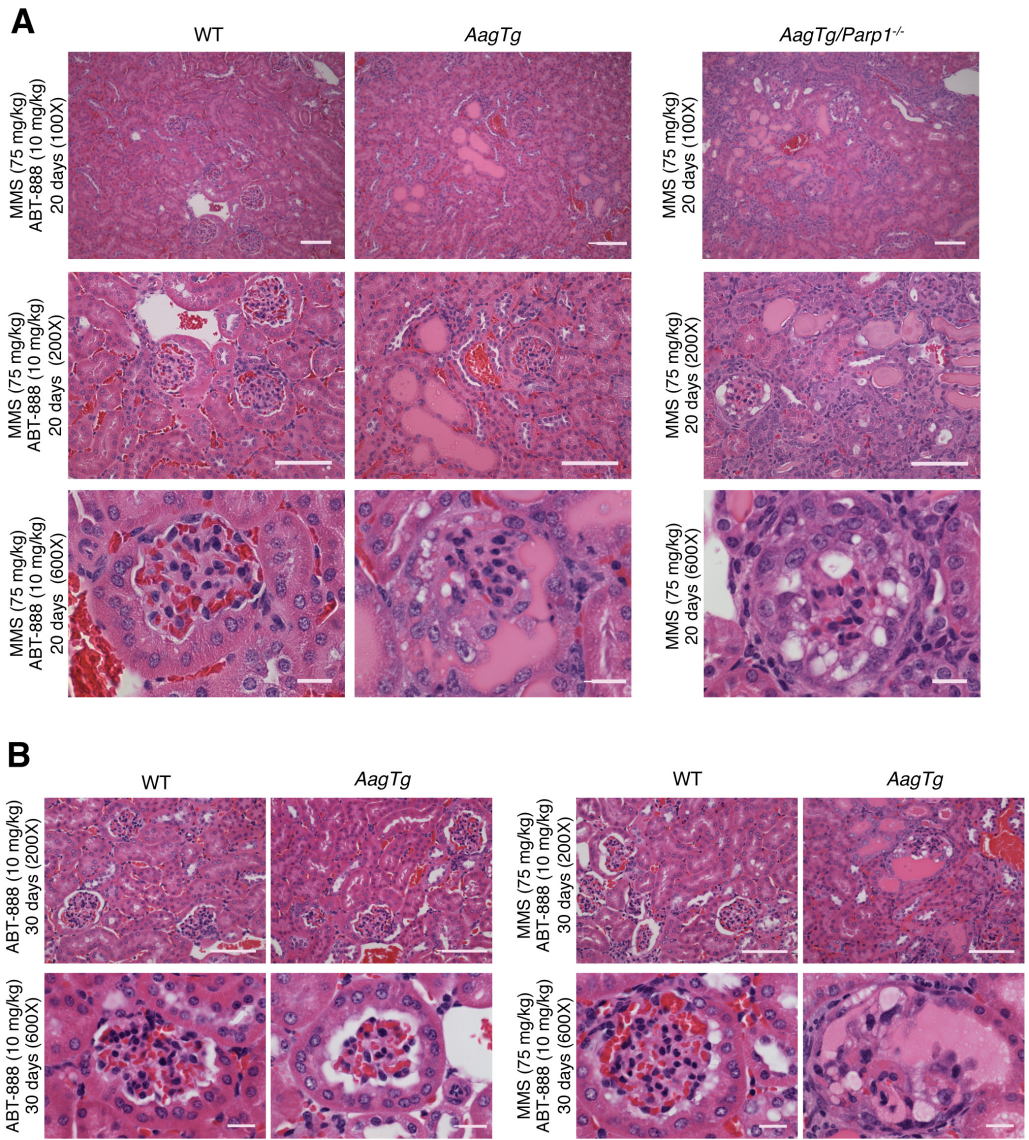
*AagTg* mice treated with a Parp inhibitor exhibit MMS-mediated renal toxicity similar to *AagTg/Parp1^−/−^* mice H&E stained images of kidneys from WT, *AagTg,* and *AagTg/Parp1^−/−^* mice 20 days (A) and 30 days (B) following MMS (75 mg/kg) and/or ABT-888 (10 mg/kg) treatment as indicated. ABT-888 was administered 1 hour prior and 5 days post-MMS treatment. Magnification is 100X, 200X (scale bar 50 μm) and 600x (scale bar 10 μm); MMS- and ABT-888-treated *AagTg* mice show islands of pink proteinaceous casts, dilation of the kidney tubules, and few glomeruli with severe abnormalities (including vacuolation and the loss of Bowman's space) as seen in *AagTg/Parp1^−/−^* mice post-MMS treatment.

*AagTg* mice treated with ABT-888 (a.k.a. Veliparib), a clinically-relevant PARP inhibitor, exhibit MMS-mediated renal toxicity similar to *AagTg/Parp1^−/−^* mice (Figure 3) indicating that Parp inhibition was sufficient to sensitize *AagTg* mouse kidneys to MMS. However, renal toxicity was reduced in MMS/ABT-888-treated *AagTg* mice compared to MMS-treated *AagTg/Parp1^−/−^* mice, with affected glomeruli and protein casts being less prevalent. Consistent with the reduced severity of the kidney phenotype, MMS/ABT-888-treated *AagTg* mice exhibited lower whole-body sensitivity compared to *AagTg/Parp1^−/−^* mice; at 20d post-MMS treatment, no *AagTg* mice were moribund and >80% of the *AagTg* mice showed no sign of distress through 30d post-MMS (not shown).

Having shown that *AagTg* mice combined with either the *Parp1^−/−^* allele or Parp inhibition are susceptible to MMS-mediated renal toxicity, we determined whether this toxicity correlates with the accumulation of BER intermediates and lack of Parp activity. The Aag glycosylase generates potentially toxic abasic (AP) sites that can block replication and transcription [18, 19], and their cleavage by AP endonuclease generates highly toxic DNA single-strand breaks [20]; indeed, until the final ligation step of BER, these toxic lesions are present in DNA. Thus, if BER is initiated under conditions where the subsequent processing of BER intermediates is limiting, i.e. by Parp deficiency, this can result in cell death and tissue damage; under these circumstances cells are said to have an imbalanced BER pathway [11]. Indeed, at 3 hours post-MMS treatment, *AagTg* kidney DNA contained twice as many AP sites as WT kidney DNA (Supplemental Figure 3A). Consistent with these findings, at 6 hours post-MMS treatment, Parp activity was highly increased in *AagTg* kidneys compared to WT kidneys (Supplemental Figure 3B). In both WT and *AagTg* kidneys, Parp activity peaks at 24 hours post-MMS treatment and returns to basal levels within 48 hours post-MMS treatment. Conversely, although *AagTg/Parp1^−/−^* kidney DNA had high levels of AP sites similarly to *AagTg* kidney DNA at 3 hours post-MMS treatment (Supplemental Figure 3A), no increase in Parp activity was observed in *AagTg/Parp1^−/−^* kidneys at any time point (Supplemental Figure 3B). These findings support the hypothesis that an imbalanced BER pathway can be detrimental for the kidney.

### Progression of MMS-mediated renal toxicity in *AagTg/Parp1^−/−^* mice

**Figure 4:**
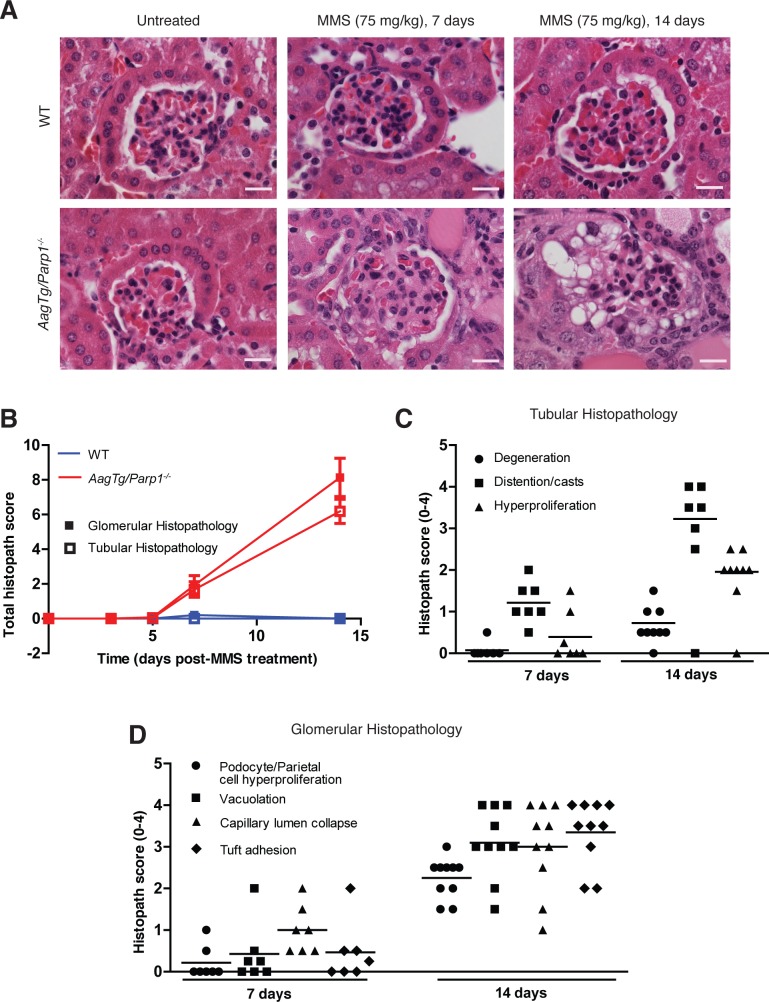
Kinetics of kidney damage in *AagTg/Parp1^−/−^* mice following MMS treatment **A.** H&E-stained images of kidneys from WT and *AagTg/Parp1^−/−^* mice under untreated conditions and 7 and 14 days following MMS treatment (75 mg/kg). Magnification is 600X (scale bar 10 μm). **B.** Total histopathology scores for glomerular damage (podocyte/parietal cell hyperplasia/hypertrophy, vacuolation, capillary lumen collapse, tuft adhesions and/or sclerosis) and tubular damage (degeneration, cast formation, hyperplasia) is shown at five time points, post MMS treatment (75 mg/kg); untreated (WT, *n* = 5 and *AagTg/Parp1^−/−^*,*n* = 7), 3 days (WT, *n* = 3 and *AagTg/Parp1^−/−^*, *n* = 2), 5 days (WT, *n* = 7 and *AagTg/Parp1^−/−^*, *n* = 10), 7 days (WT, *n* = 5 and *AagTg/Parp1^−/−^*, *n* = 7 and 14 days (WT, *n* = 10 and *AagTg/Parp1^−/−^*, *n* = 11). Histopathological scores for **C.** renal tubule criteria and **D.** glomerular criteria.

We examined the kinetics of MMS-induced kidney damage by monitoring kidney toxicity at 3, 5, 7 and 14d post-MMS. We assessed the glomerulus, renal tubules and Bowman's capsule following histopathological criteria listed in Supplemental Table 1. No histopathological abnormalities were observed at any time point in WT mice and in *AagTg/Parp1^−/−^* mice at 3d or 5d post-MMS (Figure 4A-4C). However, by 7d we observe abnormalities in *AagTg/Parp1^−/−^* kidneys. One obvious change within the renal tubules at 7d post-MMS is tubule dilation and presence of proteinaceous casts (Figure 4C). By 14d there is also evidence of reactive changes in the tubules, including mild degeneration and hyperplasia (Figure 4C). Consistent with reactive regeneration, epithelial mitotic figures are also observed (Supplemental Figure 4). The glomerulus exhibits histological abnormalities as early as 7d post-MMS, including vacuolation of podocytes or parietal cells and capillary collapse of the lumen (Figure 4D). By 14d, glomeruli in *AagTg/Parp1^−/−^* mice exhibit significant increases in the histological scores of podocyte or parietal cell hyperplasia (*p* = 0.0005), vacuolation (*p* = 0.001) and capillary lumen collapse (*p* < 0.05). At 14d post-MMS, additional glomerular pathological abnormalities appear, including crescent formation (Figure 2B and 4A), a phenomenon commonly observed in crescentic glomerulonephritis in both humans and mice [21–23].

### *AagTg/Parp1^−/−^* mice exhibit clinical markers of kidney disease

**Figure 5:**
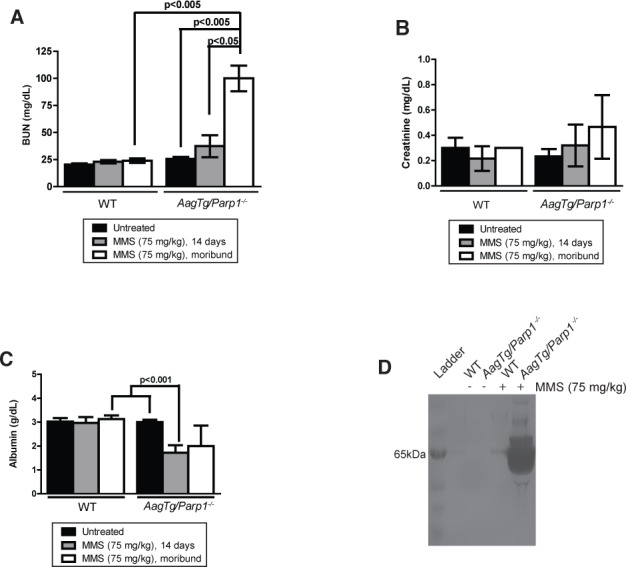
*AagTg/Parp1^−/−^* mice exhibit clinical signs of kidney damage Serum levels of **A.** blood urea nitrogen (BUN), **B.** creatinine, and **C.** albumin were measured in untreated (n = 3-4), 14 days post-MMS treatment (75 mg/kg) (*n* = 5-6), and when mice exhibit morbidity (*n* = 3). For the moribund time point, a control WT mouse was assayed with each *AagTg/Parp1^−/−^* mouse. **D.** Silver stain visualization of protein content in urine of WT and *AagTg/Parp1^−/−^* in untreated conditions and 7 days following MMS treatment (75 mg/kg).

We next investigated whether kidney abnormalities observed at the histological level were accompanied by clinical markers of disease. Serum analytes were measured before treatment, 14d post-MMS, and when the mice exhibited morbidity post-MMS. We observe a trend toward higher serum levels of blood urine nitrogen (BUN) in *AagTg/Parp1^−/−^* mice 14d post-MMS compared to WT mice (*p* = 0.157) (Figure 5A); this trend became significant in moribund *AagTg/Parp1^−/−^* mice (*p* < 0.005) (Figure 5A). Levels of blood creatinine, another marker of kidney damage, did not change significantly post-MMS treatment (Figure 5B).

Serum albumin levels, a marker of glomerular function, displayed dramatic decreases following MMS in *AagTg/Parp1^−/−^* mice compared to treated WT mice or untreated *AagTg/Parp1^−/−^* mice (Figure 5C). In addition, we observed high levels of a ~65 kDa protein, consistent with the size of albumin, in the *AagTg/Parp1^−/−^* mouse urine 7d post-MMS (Figure 5D). Together, these data indicate that albumin is not properly maintained in the serum, but is likely accumulating in the kidney tubules as proteinaceous casts, and ultimately leaking into the urine of MMS-treated *AagTg/Parp1^−/−^* mice. The albumin leakage in the urine 7d post-MMS is consistent with histopathological changes within the glomerulus observed at the same time point (Figure 5D). These features indicate impaired glomerular function induced by MMS.

### *AagTg/Parp1^−/−^* mice exhibit MMS-induced podocyte injury

**Figure 6:**
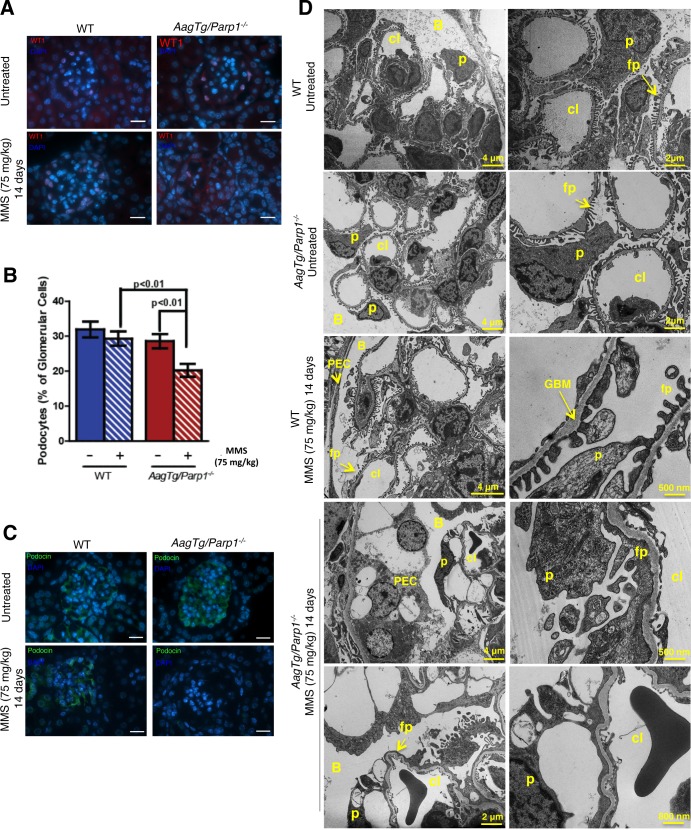
Podocyte damage is observed following MMS treatment in *AagTg/Parp1^−/−^* mice **A.** Immunofluorescence staining of WT1-positive podocytes in WT and *AagTg/Parp1^−/−^* mice, untreated and 14 days following MMS treatment (75 mg/kg). A representative image of *n* = 3 mice is shown. Magnification is 600X (scale bar 10 μm). **B.** Quantitation of podocytes (the % of cells stained with both WT1 and DAPI/total number of DAPI positive cells in the glomerulus) is shown. Eight glomeruli were counted on each section from *n* = 3-4 mice/condition. **C.** Immunofluorescence staining of Podocin in WT and *AagTg/Parp1^−/−^* mice in untreated and 14 days following MMS treatment (75 mg/kg). Magnification is 600X (scale bar 10 μm). **D.** Transmission electron microscopy (TEM) of WT and *AagTg/Parp1^−/−^* mice, untreated and 14 days following MMS treatment (75 mg/kg). P, podocytes; fp, foot processes; cl, capillary lumen; B, Bowman's capsule; GBM, glomerular basement membrane; PEC, parietal epithelial cell.

Given the apparent loss of glomerular filtration, we examined whether the *AagTg/Parp1^−/−^* mice displayed podocyte injury post-MMS by immunostaining for Wilm's tumor 1 (WT1) protein, a podocyte marker. MMS-treated *AagTg/Parp1^−/−^* mouse kidneys exhibited a decreased number of WT1-stained cells. No significant change was observed in MMS-treated WT mice (Figure 6A-6B). Furthermore, we investigated a potential alteration in the slit diaphragm by immunostaining for Podocin. MMS-treated *AagTg/Parp1^−/−^* mice exhibit diminished staining of Podocin, consistent with reduced glomerular filtration (Figure 6C).

Electron-microscopy (Figure 6D) revealed that WT mice (untreated and14d post-MMS) had normal glomerular tuft architecture comprised of well-organized capillary loops, Bowman's capsule and flat parietal epithelial cells (PECs). Podocytes looked healthy with numerous well-organized foot processes. Untreated *AagTg/Parp1^−/−^* mouse kidneys appeared similar to the WT controls in ultrastructural morphology of glomerular tufts. However, MMS-treated *AagTg/Parp1^−/−^* mouse kidneys (14d) showed ultrastructural alterations consistent with the main histological alterations observed in the podocytes and PECs. As shown in Figure 6D, PECs and podocytes were markedly hypertrophied by the presence of multiple intracytoplasmic medium-to-large-sized membrane-bound vacuoles. These vacuoles were mostly empty or occasionally contained pale to electron-dense irregular granular to amorphous material. The capillary tufts were distorted and compressed of numerous large distinct vacuoles within the podocyte foot processes. Extensive flattening and alteration of podocyte foot processes was observed. There was also a discernible reduction in the area of capillary loop and fusion between the large vacuolated PECs and podocytes at the urinary pole, resulting in loss of urinary space. These observations confirm that *AagTg/Parp1^−/−^* mice exhibit severe morphological and functional podocyte damage post-MMS.

### Female *AagTg/Parp1^−/−^* mice are protected from MMS-induced nephrotoxicity

**Figure 7:**
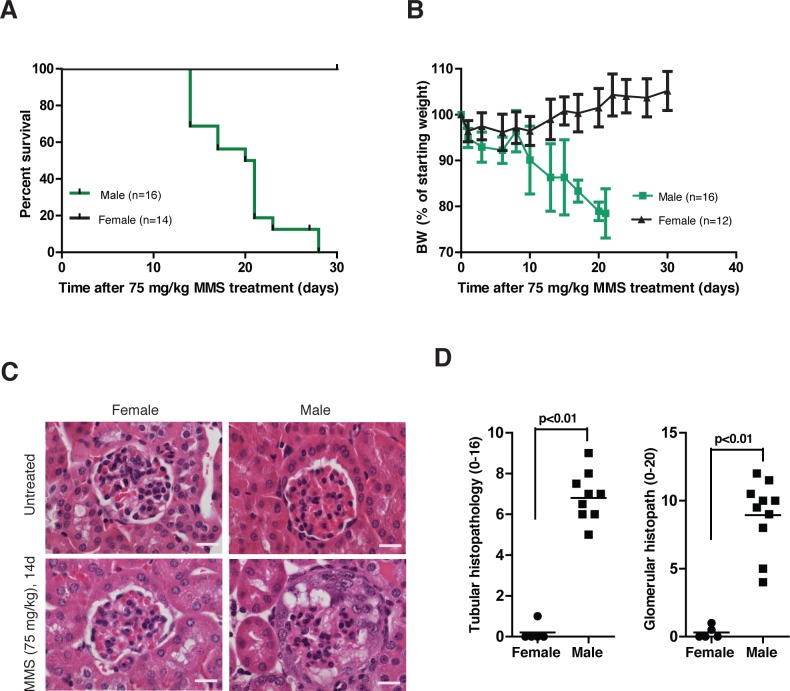
Female *AagTg/Parp1^−/−^* mice are protected from MMS-mediated kidney damage **A.** Kaplan Meier survival curves are shown for female (*n* = 14) and male (*n* = 16) *AagTg/Parp1^−/−^* mice following treatment with MMS (75 mg/kg). **B.** BW, as percent of initial BW, is shown for female *AagTg/Parp1^−/−^* (*n* = 14) and male *AagTg/Parp1^−/−^* (*n* = 16) mice following treatment with MMS (75 mg/kg). **C.** H&E-stained images from kidneys are shown for female and male *AagTg/Parp1^−/−^* mice 14 days following MMS treatment (75 mg/kg). Magnification is 600x (scale bar 10 μm). **D.** Total histopathology scores for glomerular damage (podocyte/parietal cell hyperplasia/hypertrophy, vacuolation, capillary lumen collapse, tuft adhesions/sclerosis) and tubular damage (degeneration, cast formation, hyperplasia) is shown for female and male mice 14 days post-MMS treatment (75 mg/kg). Scores in a *AagTg/Parp1^−/−^* male is also presented in Figure 4B-4D.

An unexpected sexual dimorphism became apparent in MMS-treated female *AagTg/Parp1^−/−^* mice. MMS-induced whole-animal toxicity was reduced in *AagTg/Parp1^−/−^* female mice. The MMS LD_50_ for *AagTg/Parp1^−/−^* female mice is 80 mg/kg, whereas that for *AagTg/Parp1^−/−^* male mice is 66 mg/kg. Furthermore, in contrast with the morbidity observed in male *AagTg/Parp1^−/−^* mice, female *AagTg/Parp1^−/−^* mice exhibited no body weight decrease and survived 30d post-MMS (75 mg/kg, Figure 7A-7B). Histopathological analysis confirmed complete protection against kidney damage in MMS-treated female *versus* male *AagTg/Parp1^−/−^* mice (Figure 7C-7D). The renoprotective roles of estrogen are well-established in both mice [24, 25] and humans [26, 27]; our data suggest that estrogen may also be protective against alkylation-induced nephrotoxicity in *AagTg/Parp1^−/−^* mice. Indeed, chronic 17b-estradiol treatment (E2 pellets) completely protected 6 out of 8 male mice against MMS-induced kidney damage (Supplemental Figure 5). The remaining 2 mice showed only a mild pathology with few alterations of tubules and few affected glomeruli. Moreover, MMS-mediated nephrotoxicity in males is not a consequence of the genetic background or strain, as *AagTg/Parp1^−/−^* mice on a pure 129S background exhibit kidney functional abnormalities similar to that of *AagTg/Parp1^−/−^* mice on a C57Bl/6J:129S mixed background (Supplemental Figure 6). This finding is especially relevant because genetic background has previously been shown to modify susceptibility to glomerular disorders [28, 29].

## DISCUSSION

Although protected from MMS-induced toxicity in a variety of tissues, *AagTg/Parp1^−/−^* mice eventually succumb to whole-animal lethality at lower MMS doses in comparison to *AagTg* mice. This increased lethality was dependent on imbalanced BER, caused by increased Aag levels, since the MMS LD_50_ in *Parp1^−/−^* mice expressing normal Aag levels is equivalent to that in WT mice. Interestingly, when BER is imbalanced by increased Aag activity, there are obvious tissue-specific differences with respect to whether Parp1 deficiency suppresses or exacerbates toxicity. Parp1 deficiency protects *AagTg* mice against MMS-induced tissue damage in the cerebellum, pancreas, spleen and retina [12], but enhances MMS-induced kidney damage. We examined a multitude of tissues in *AagTg/Parp1^−/−^* mice post-MMS and only found histological abnormalities in the kidney. It is possible that with time, additional tissues could display similar damage and that the rapid development of kidney disease prevents observation of such damage. Controlling Aag expression in a tissue-specific manner will help to determine whether other tissues exhibit MMS-mediated toxicity in the absence of Parp1 at longer time points. Neither increased Aag activity, nor Parp1 deficiency alone resulted in susceptibility to kidney damage, but rather the combination of both gave rise to this severe kidney phenotype post-MMS treatment. We found that *AagTg/Parp1^−/−^* mice as well as *AagTg* mice generate more BER intermediates, i.e. AP sites, than WT mice post-MMS treatment. However, in *AagTg* mice, the higher accumulation of AP sites is compensated by higher levels of Parp activity compared to WT. In both, WT and *AagTg* mice, Parp activity returns to basal levels within 48 hours when all lesions have likely been repaired. Unlike *AagTg* mice, *AagTg/Parp1^−/−^* mice do not show any Parp activity, suggesting that Parp deficiency is a limiting step in subsequent processing of BER intermediates and that the accumulation of BER intermediates, due to increased Aag activity and Parp deficiency, might be the cause of kidney toxicity. Our results are especially relevant given the fact that AAG levels can vary >10-fold in the human population (at least in lymphocytes) [12, 16, 17] and given the recent surge in the use of PARP inhibitors in combination with chemotherapeutic agents. As nephrotoxicity can be the rate-limiting side effect in a subset of chemotherapeutic agents, these findings may have important clinical relevance [30].

In contrast to our finding that MMS induces nephrotoxicity in *Parp1^−/−^* and Parp-inhibited *AagTg* mice, several other models of kidney damage have demonstrated protection under conditions of genetic or pharmacologic Parp depletion. Parp1 depletion protects against nephrotoxicity induced by the chemotherapeutic agent, cisplatin [31, 32], diabetic nephropathy [33, 34] and the necrosis associated with renal ischemia/reperfusion [35, 36]. The MMS-induced nephrotoxicity in *AagTg/Parp1^−/−^* mice was unexpected and to our knowledge has not been previously described. This finding suggests that Parp1 deletion or Parp inhibition may not give rise to the same outcomes across all tissues and experimental models, making it increasingly important to consider the clinical consequences of PARP inhibition in cancer treatment.

Parp1 is a multi-functional protein, exhibiting many functions independent of its role in DNA repair [37]. It is possible that a DNA repair-independent Parp1 function contributes to the phenotype described here. For example, Parp1 plays a role in modulating inflammation [38, 39] and although there is no evidence of altered immune cell recruitment to the glomeruli, it remains possible that immune system changes contribute to this severe glomerular phenotype. Indeed, autoimmune disorders, such as lupus nephritis, routinely result in glomerular lesions with some of the characteristics we observe [40].

Strikingly, female *AagTg/Parp1^−/−^* mice were completely protected from MMS-mediated nephrotoxicity. Males (in both human and animal models) are more prone to kidney disease [41, 42]. Indeed, epidemiological studies identified male sex as a risk factor for kidney disease [43]. Consistent with this, female mice are protected from ischemic renal injury and diabetic nephropathy, and estrogen provides protection against ischemia-reperfusion renal injury and age-related kidney decline [44–46], but does not protect against cisplatin-mediated nephrotoxicity [47]. Specifically, stimulation of estrogen receptors protects against podocyte damage and death both *in vitro* and *in vivo* [48, 49]. Not only is estrogen protective, but testosterone potentiates renal injury [50–53]. Here, we show that estrogen treatment protects male mice from alkylation-induced nephrotoxicity. These findings further support the role of estrogen in protection against kidney disease. However, in addition to sex steroids, sexual dimorphisms in renal morphology and physiology [54, 55] may also contribute to the protection of female *AagTg/Parp1^−/−^* mice from MMS-mediated kidney disease. Additionally, it has been described previously that female and male mice exhibit different responses to Parp1 deletion or Parp inhibition in mouse models of endotoxemia and nephrotoxic serum-induced nephritis [56, 57]. These data further underscore the recent push for investigating and understanding sex differences in basic and clinical science [58–61].

The sexual dimorphism in our model may be explained by the interaction of Aag and Parp1 with estrogen receptor-α (ERα) [62, 63]. A direct interaction between Aag and ERα has been described; binding of Aag and ERα causes increased catalytic activity of Aag and decreased ERα-mediated transcription [62]. Parp1 has been shown to bind and PARylate ERα, leading to increased binding of ERα to the estrogen response element in the promoter of target genes, thus promoting ERα-mediated transcription [63]. Together, these findings suggest that a direct interaction between Aag and/or Parp1 with estrogen receptors could lead to changes in DNA repair and/or gene expression, explaining the sexual dimorphism of MMS-mediated nephrotoxicity in mice.

Numerous existing models of acquired podocyte diseases are used to explore human renal diseases, however all models have both advantages and disadvantages [14]. We present here a new model that may provide insight into the mechanisms of podocyte injury and death following treatment with alkylating agents.

## MATERIALS AND METHODS

### Animals and treatments

*Parp1^−/−^* mice were purchased from Jackson Laboratories [64]. *Aag* transgenic (*AagTg*) mice were described previously [12, 13, 65]. All experiments were performed in mixed background mice (C57Bl/6J:129S) unless otherwise stated. All animal procedures were performed according the NIH guide for the Care and Use of Laboratory Animals.

Approximate LD_50_ was determined as in Deichmann and LeBlanc [66]. MMS was injected intraperitoneally. ABT-888 (10 mg/kg, Selleck Chemicals Inc) was administered by oral gavage 1 hour before and 5 days after MMS treatment. For estrogen treatment, 17b-estradiol pellets (E2, 0.1 mg/21 days release, Innovative Research of America) were implanted s.c. in the dorsal neck region of adult male mice. MMS was administered 3 days after implants.

### AP sites and PARP activity measurement

DNA was isolated using Roche kit according to the manufacturer's instructions with precautions taken to avoid overheating of the DNA solution (proteinase K step 10 min at 55°C). AP sites were determined by using the DNA Damage Quantification Kit (Dojindo) and performed according to the manufacturer's instructions. Parp activity was determined by using the HT PARP *in vivo* Pharmacodynamic Assay II (Trevigen) according to the manufacturer's instructions.

### Serum analytes and albumin gel electrophoresis

Whole blood was collected *via* cardiac puncture and placed in BD microtainer serum separator tubes (VWR). Blood samples were then centrifuged for 5 minutes at 2000 x g to separate serum. Idexx Laboratories, Inc. (North Grafton, MA) performed serum chemistry analysis.

Two μL of urine was denatured and run on a SDS-PAGE gel and proteins were visualized using ProteoSilver Plus Silver Staining Kit (Sigma).

### Electron microscopy

Tissue processing and imaging was performed by the W.M. Keck Microscopy Facility at the Whitehead Institute (MIT). Kidney cortex was fixed in 2.5% gluteraldehyde, 3% paraformaldehyde with 5% sucrose in 0.1 M sodium cacodylate buffer (pH 7.4), post-fixed in 1% osmium tetroxidein veronal-acetate buffer, stained in block overnight with 0.5% uranyl acetate in veronal-acetate buffer (pH 6.0) and then embedded in Embed-812 resin. Ultrathin sections were cut from blocks on a Leica Ultracut UCT microtome with a Diatome diamond knife. The sections were examined using a FEI Tecnai Spirit at 80 keV.

### Histopathology

Tissues were processed by the Histology Core Facility at the David H. Koch Institute for Integrative Cancer Research (MIT); they were paraffin-embedded, sectioned at 5 μm, and stained with hematoxylin and eosin (H&E), or Periodic Acid-Schiff (PAS). Phosphotungstic acid-haematoxylin (PTAH) staining was performed at the Division of Comparative Medicine (MIT). All H&E stained slides were blindly analyzed by a pathologist (R.T.B). Renal lesions were blindly scored by a pathologist (S.M.) using criteria listed in Supplemental Table 1.

### Immunofluorescence

Deparaffinized tissue sections (5 μm) were thermally processed for epitope retrieval using a Prestige Medical 2100-Retriver. Sections were permeabilized in PBS-T (1x PBS + 0.1% Triton X-100, three times for 5 minutes each), incubated with 1x PBS-T plus 10% goat serum for 1 hour and then stained with either anti-WT1antibody (Santa Cruz) or anti-podocin (Sigma) overnight at 4°C. Nuclear counterstaining was done using DAPI. The number of glomerular DAPI- and WT1-stained cells were counted using ImageJ.

### Statistics

Statistical analyses were performed using GraphPad Prism software. Statistical significance was determined using unpaired t-test. Kaplan-Meier survival curves were generated and survival differences determined using the Log-Rank test. Data are represented as mean +/− SEM. A p-value is considered significant if less than 0.05.

## SUPPLEMENTARY MATERIALS FIGURES AND TABLES


