# Supplementary material for: Parp1 protects against Aag-dependent alkylation-induced nephrotoxicity in a sex-dependent manner
Source: Oncotarget. 2016 Jul 6;7(29):44950–65. doi: 10.18632/oncotarget.10440 (PMC5216697; doi:10.18632/oncotarget.10440)
Supplement: Supplementary file 1 [file oncotarget-07-44950-s001.pdf]

Parp1 protects against Aag-dependent alkylation-induced nephrotoxicity in a sex-dependent manner

Supplementary Material

Contents:

Supplemental Table 1. Renal histopathology scoring criteria.

Supplemental Figure 1. *AagTg/Parp1$^{-/-}$* kidneys exhibit collapse of the glomerular tuft.

Supplemental Figure 2. *AagTg/Parp1$^{-/-}$* kidneys show no evidence of fibrin thrombi nor staining of vacuoles.

Supplemental Figure 3. *AagTg/Parp1$^{-/-}$* kidneys have an imbalanced BER pathway.

Supplemental Figure 4. *AagTg/Parp1$^{-/-}$* kidneys exhibit mitotic figures in tubules and Bowman's capsule.

Supplemental Figure 5. Chronic E2 treatment rescues MMS-induced kidney damage in *AagTg/Parp1$^{-/-}$* male mice.

Supplemental Figure 6. *AagTg/Parp1$^{-/-}$* mice on 129S background exhibit similar kidney disease as *AagTg/Parp1$^{-/-}$* mice on C57Bl/6J:129S background.

Supplemental Table 1: Renal histopathology scoring criteria.

**Glomerular alterations: Semi- quantitative scoring**		
1.	Inflammation (0-4): none, minimal, mild, moderate, severe	
2.	Glomerular hyperplasia/hypertrophy (0-4): none, minimal, mild, moderate, severe<br>Mitosis if present is noted.	
	a. Podocyte and/or Parietal cell hyperplasia/hypertrophy	b. Mesangial hyperplasia/hypertrophy
3.	Vacuolation (0-4): Presence of distinct vacuoles in glomeruli within any of the following components : mesangium, visceral epithelial cells (podocytes), and parietal epithelial cells	
4.	Capillary lumen and mesangial collapse and/or obliteration (0-4): none, minimal, mild, moderate, severe	
5.	Glomerular Tuft adhesions and/or Sclerosis(0-4): none, minimal, mild, moderate, severe	
	a. Glomerular tuft adhesions: Presence of synechiae between glomerular tufts and Bowman's capsule	b. Glomerular sclerosis: Increased thickness of capillary walls, mesangium and basement membranes by fibrous connective tissue matrix
6.	Glomerular necrosis/dissolution (0-4): none, minimal, mild, moderate, severe	
**Tubular changes: Semi- quantitative scoring and qualitative evaluation**		
1.	Inflammation (0-4): None, minimal, mild, moderate, severe	
2.	Degeneration/Necrosis(0-4): None, minimal, mild, moderate, severe	
3.	Distension/Cast formation(0-4): None, minimal, mild, moderate, severe	
4.	Hyperplasia/Regeneration(0-4): None, minimal, mild, moderate, severe	
5.	Dysplasia – noted if present, no scoring	
**Interstitial changes- Semi- quantitative scoring**		
1.	Inflammation(0-4): None, minimal, mild, moderate, severe	
2.	Fibrosis(0-4): None, minimal, mild, moderate, severe	
**Vascular alterations: Qualitative evaluation**		
1.	Vasculitis: Noted if present	
2.	Fibrin Thrombi: Noted and graded on severity of distribution if present in glomerular capillary and/or interstitial vasculature	

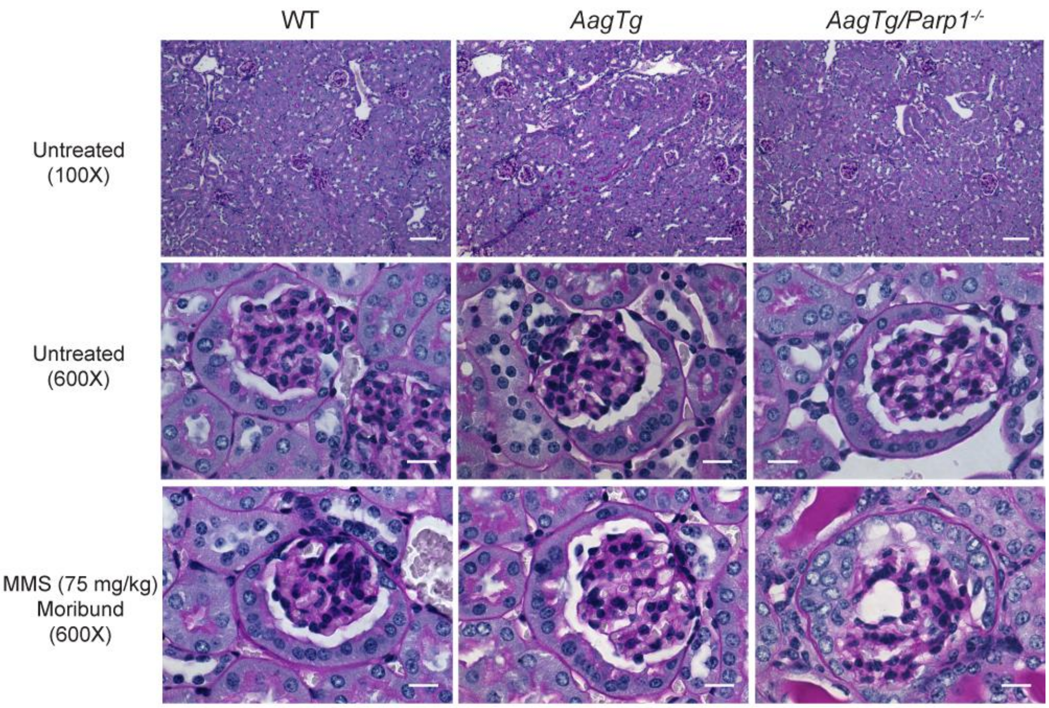

Supplemental Figure 1. ***AagTg/Parp1$^{-/-}$*** **kidneys exhibit collapse of the glomerular tuft.**
PAS stained images of kidneys from WT, *AagTg*, and *AagTg/Parp1$^{-/-}$* mice, untreated and 14 days following MMS treatment (75 mg/kg). Magnification is 100X (scale bar 75 µm) and 600x (scale bar 15 µm). Untreated and MMS-treated WT and *AagTg* mice and untreated *AagTg/Parp1$^{-/-}$* mice show healthy glomerular tuft with discernible capillary loop outlines with luminal red blood cell profiles; MMS-treated *AagTg/Parp1$^{-/-}$* mice show disorganized collapsed glomerular capillary tufts surrounding large unstained vacuoles and hyperplastic/hypertrophic capsular epithelial cells.

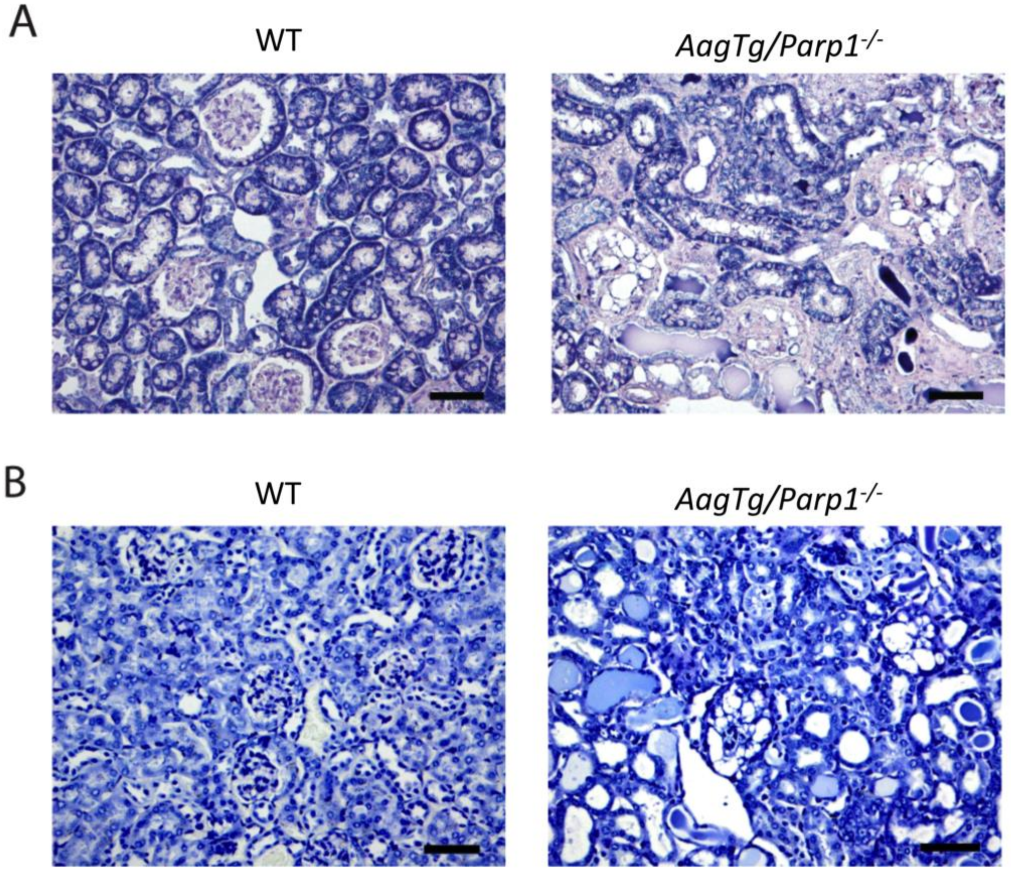

Supplemental Figure 2. ***AagTg/Parp1*$^{-/-}$ kidneys show no evidence of fibrin thrombi nor staining of vacuoles.**

(A) PTAH- and (B) Toludine blue-stained kidneys from WT and *AagTg/Parp1*$^{-/-}$ mice 14 days following MMS treatment (75 mg/kg); Magnification 400X (scale bar 50 µm).

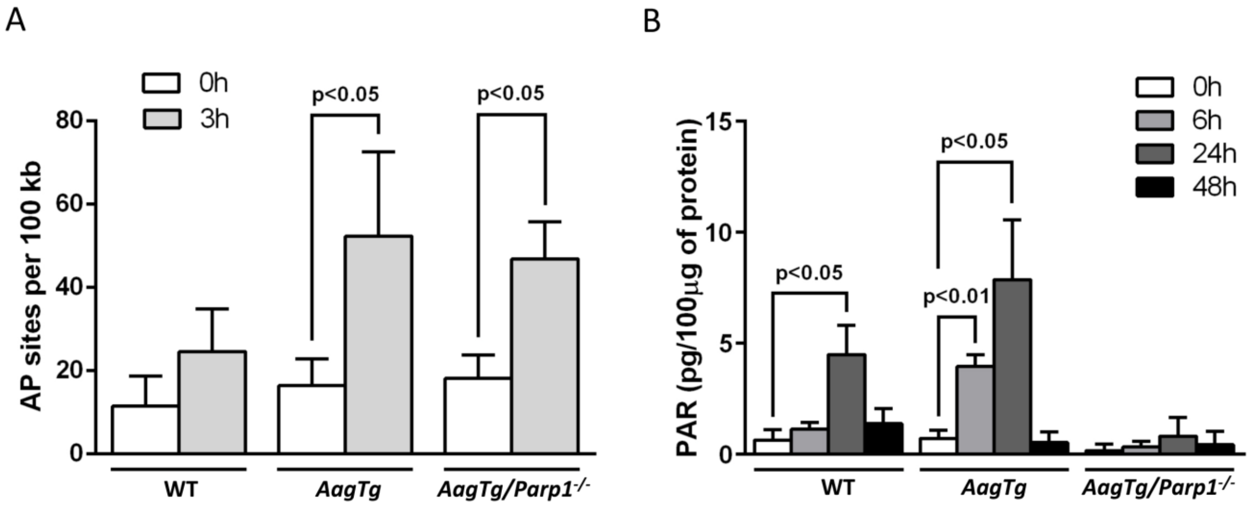

Supplemental Figure 3. *AagTg/Parp1$^{-/-}$* kidneys have an imbalanced BER pathway. (A) Levels of abasic (AP) sites at 3 hours post-MMS treatment in kidney DNA isolated from WT, *AagTg* and *AagTg/Parp1$^{-/-}$* mice. (B) Parp activity in WT, *AagTg* and *AagTg/Parp1$^{-/-}$* kidneys at 0, 6, 24 and 48 hours post-MMS treatment. Parp activity is represented as pg of polymer poly-ADP-ribose (PAR) generated for 100 μg of protein (n=3-5).

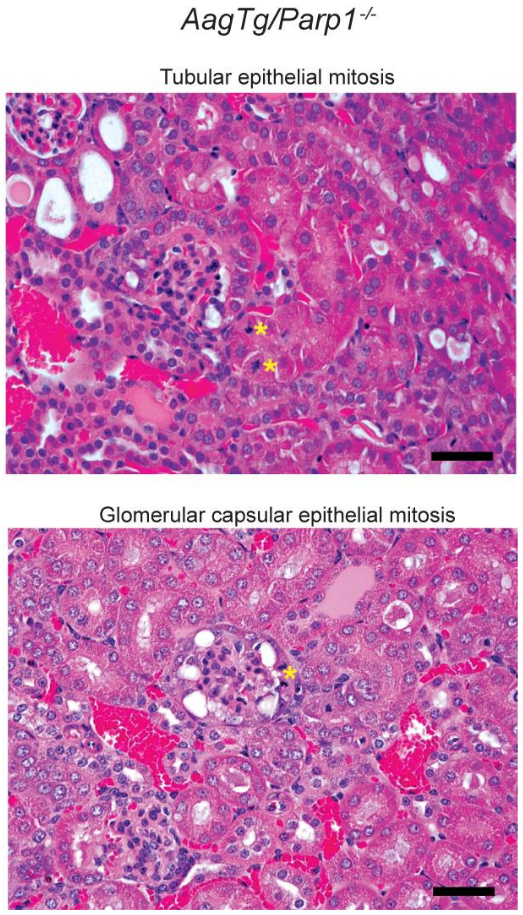

Supplemental Figure 4. ***AagTg/Parp1**$^{-/-}$* **kidneys exhibit mitotic figures in tubules and Bowman's capsule.**

H&E-stained images from *AagTg/Parp1*$^{-/-}$ mouse kidneys when *AagTg/Parp1*$^{-/-}$ mice become moribund following MMS treatment (75 mg/kg). Magnification is 200X (scale bar 50 μm); yellow asterisks indicates mitotic figures.

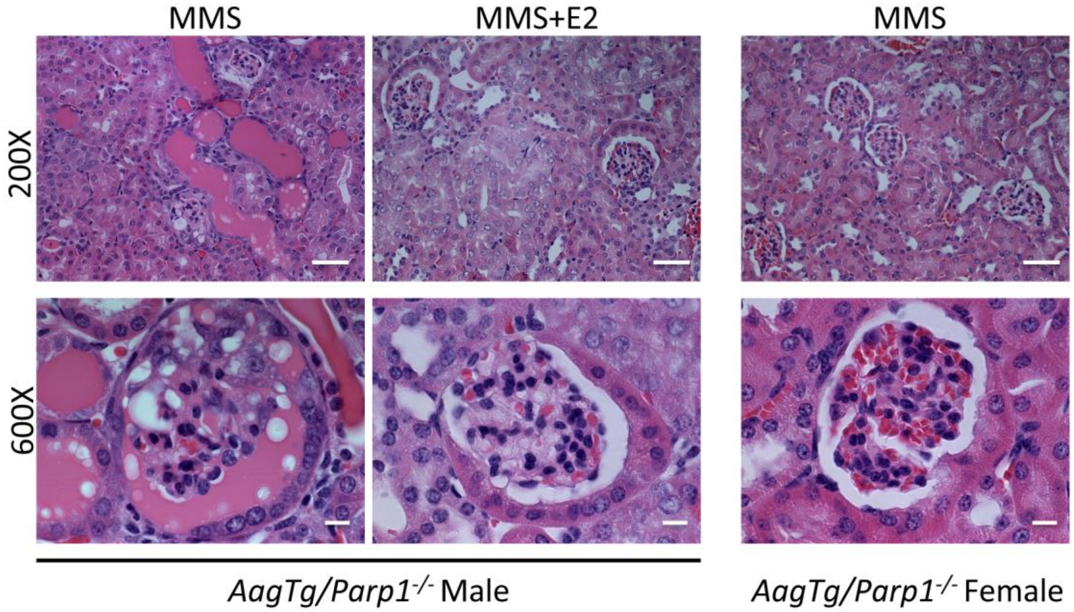

Supplemental Figure 5. Chronic E2 treatment rescues MMS-induced kidney damage in *AagTg/Parp1*$^{-/-}$ male mice. H&E stained images from *AagTg/Parp1*$^{-/-}$ male and female mouse kidneys 14 days following MMS treatment (75 mg/kg) and E2 treatment (0.1 mg/pellet) as indicated. Magnification 200X (scale bar 50 µm) and 600X (scale bar 10 µm.).

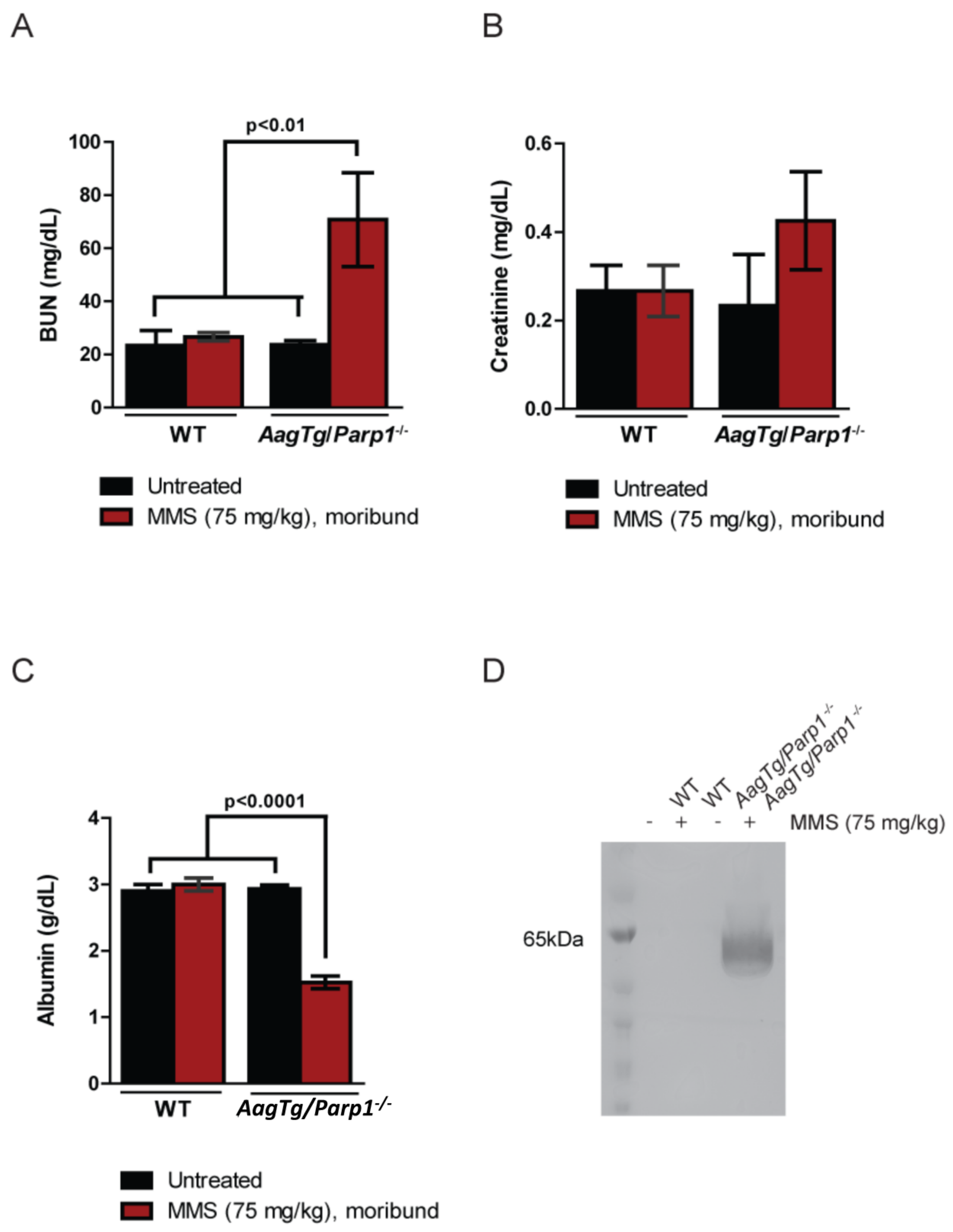

Supplemental Figure 6. ***AagTg/Parp1**$^{-/-}$* **mice on 129S background exhibit similar kidney disease as *AagTg/Parp1*$^{-/-}$ mice on C57Bl/6J:129S background.**

Serum levels of (A) blood urea nitrogen (BUN), (B) creatinine, and (C) albumin were measured in untreated (n=3) and when mice exhibit morbidity post MMS-treatment (75 mg/kg, n=4). For the moribund time point, a control WT mouse was assayed with each *AagTg/Parp1*$^{-/-}$ mouse. (D) Silver stain visualization of protein content in urine of WT and *AagTg/Parp1*$^{-/-}$ in untreated conditions and 7d following MMS treatment (75 mg/kg). All the mice used in this figure are on pure 129S1 background. Note the significant increases in BUN (A, p<0.01), a trend towards increased creatinine (B, p=0.23), decreased serum albumin (C, p<0.0001), and albumin leaking into the urine (D) in *AagTg/Parp1*$^{-/-}$mice following MMS treatment.
